# Ambivalent Tolman: Indirect Influence on Enactivism of Tolman's Sign‐Gestaltism Through Merleau‐Ponty

**DOI:** 10.1002/jhbs.70053

**Published:** 2026-04-13

**Authors:** Hiroshi Matsui, Kohei Yanagawa

**Affiliations:** ^1^ Graduate School of Human Sciences The University of Osaka Suita Japan; ^2^ Kinugasa Research Organization Ritsumeikan University Kyoto Japan

## Abstract

Edward Tolman, an experimental psychologist renowned for his research on the “cognitive map” and “latent learning” in rats, pursued his career within the tradition of behaviorisms. In the history of psychology, he is positioned as a precursor to cognitivism. This is because the concepts he introduced as intervening variables were later interpreted as having a representational nature. On the other hand, the philosophy of Maurice Merleau‐Ponty was also influenced by Tolman's concepts, particularly his theory of sign‐Gestalt. Merleau‐Ponty's ideas, especially as developed in *The Structure of Behavior*, have had a profound impact on the notion of embodiment within enactivism. Enactivism, as a school of thought opposing representationalism, sharply contrasts with cognitivism, which places Tolman at the intersection of two opposing intellectual currents. This paper, thus, reexamines Tolman's ambivalent nature, exploring how it arose and reassessing his ideas as a precursor not only to cognitivism but also *indirectly* to enactivism. We argue that Merleau‐Ponty inherited Tolman's concept of sign‐Gestalt as having a non‐representational nature and utilized it within his relational account of behavior. While Tolman's own theoretical framework remained ontologically ambiguous, Merleau‐Ponty recast his insights into a conception of behavior as a structured, embodied interaction between organism and environment. These reformulated insights subsequently provided a conceptual foundation for enactivist cognitive science, particularly in its emphasis on the co‐constitution of perception and action, and the dynamic coupling of agent and environment. Tolman maintained a behaviorist outlook throughout his early career, but his writing often remained ambiguous; as a result, that ambiguity enabled his work to exert influence on two opposing intellectual traditions at once.

## 1 Introduction

Enactivism is a recent philosophical trend in cognitive science that emerged as a critique of classical cognitivism (Gallagher 2023). The central doctrines of cognitivism are computationalism and representationalism (e.g., Pylyshyn 1980; Thompson 2007, 4–8). In this view, cognition is understood as internally formed representations, and cognition refers to the processes of transforming and manipulating these representations through information processing. In contrast, enactivism explicitly adopts an anti‐representational stance. Although enactivism is not monolithic (Ward et al. 2017), it is consistent on this point. Enactivism holds that cognition is constituted by an organism's embodied engagement with its environment (Gallagher 2008; Varela et al. 1991). For instance, on a traditional cognitivist view, social interaction is understood as a process of inference about others, mediated by mental models such as a theory of mind. Gallagher (2008), however, argued against this picture. For him, another person's intentions are directly perceived in the other's dynamic action, taken together with the surrounding situation (i.e., direct social perception).

At the core of enactivism is the idea that cognition is embodied. Contemporary embodied cognition can be divided into weak embodied cognition and strong embodied cognition, depending on the emphasis placed on this claim (Gallagher 2017, 2023). Weak embodied cognition emphasizes the role of body representation in cognition. This perspective retains the representationalism inherent in cognitivism. In contrast, strong embodied cognition holds that cognition is *constituted* by embodied action and ongoing interaction with the environment; on this view, the body is not merely an output apparatus but plays a substantive role in explaining cognition. In this view, cognition is not seen as the reconstitution of the external world through representations, nor as information processing involving the manipulation of those representations. Enactivism aligns with the latter perspective. Merleau‐Ponty's phenomenology is often cited as a precursor to this form of embodied cognition (Thompson 2007; Gallagher 2017). While Merleau‐Ponty's influence is evident throughout enactivism, it is particularly notable in the action‐oriented view of cognition.

We argue that Merleau‐Ponty was significantly influenced by Edward Tolman in key aspects of his thought. Tolman, along with Burrhus Frederic Skinner and Clark Hull, is a prominent psychologist associated with the so‐called “neo‐behaviorist” movement since the 1930s. His purposive behaviorism, or sign‐Gestaltism, is often discussed as part of post‐Watsonian behaviorism. Tolman criticized Watson, arguing that the behavior of organisms cannot be explained merely by stimulus‐response chains (Tolman 1932). This rejection of reducing behavior to stimulus‐response sequences was heavily influenced by Gestalt psychology. One of Tolman's famous conceptual tools is the idea of the cognitive map. His work is highly regarded in cognitive science for anticipating many aspects of cognitivism (e.g., Amundson 1986; Bermúdez 2014). It is well known that Merleau‐Ponty was influenced by Gestalt psychology. Although Embree (1980) has provided a detailed analysis of this influence, Tolman was only mentioned in a single sentence. However, as we will demonstrate in a later section, Merleau‐Ponty frequently referred to Tolman in his works and adopted concepts developed by him in his own philosophy. Given these, it remains unclear how Merleau‐Ponty specifically engaged with Tolman's ideas.

Although enactivism, strongly influenced by Merleau‐Ponty, sharply contrasts with cognitivism, tracing the intellectual origins of both reveals a shared source in Tolman's thought. This raises a question that we will address in the present article: How did Tolman influence two seemingly contradictory intellectual movements simultaneously? The prevailing view of Tolman has focused primarily on his affinities with cognitivism, leaving his “ambivalence” largely unexamined.

This paper, thus, aims to explore the reception of Tolman's sign‐Gestaltism in Merleau‐Ponty and identify the underlying connections to enactivism. The paper reinterprets Tolman's legacy, situating him not only as a precursor to cognitivism but also as a hidden source of enactivism. This endeavor reveals the ambivalence inherent in Tolman's thought, serving as a source that simultaneously connects two seemingly opposing intellectual movements. To clarify this ambivalence, the paper proceeds in three steps. First, it analyzes Tolman's purposive behaviorism and his concept of sign‐Gestalt, with special attention to the ontological ambiguity surrounding the status of sign‐Gestalt (Sections 2 and 3). Second, it examines how Merleau‐Ponty received and reformulated these ideas in *The Structure of Behavior*, both drawing from and critically reworking Tolman's concepts (Section 4). Third, the paper argues that this philosophical mediation laid the conceptual groundwork for key aspects of enactivist cognitive science (Section 5). Finally, we situate the present portrayal of Tolman within the broader range of prior interpretations (Section 6). In doing so, we reassess Tolman not only as a precursor to classical cognitivism, but also indirectly as a hidden source of enactivism.

## 2 Overview of Sign‐Gestaltism

Tolman's thought emerged as a critique of Watson's original behaviorism (Tolman 1932). Therefore, it is necessary to briefly review Watson's behaviorism. Watson is widely recognized as the founder of classical behaviorism (Watson 1913a). While Watson's views significantly changed throughout his career, three key points remain crucial in understanding his stance. First, Watson limited the subject matter of psychology to observable behavior. This well‐known aspect of his argument is often referred to as *methodological behaviorism* (Morris and Todd 1999). Second, Watson's concept of “behavior” as the subject of scientific inquiry contrasts with Tolman's one. Although Watson claimed that the subject matter of psychology is behavior of the whole organism (Watson 1925, p. 14), he often explained behavior by reducing it to microscopic muscular and glandular activities in a stimulus‐response chain (e.g., Watson 1924).

A third characteristic is also notable for comparing Watson's behaviorism with Tolman's: a treatment of consciousness and mental processes. It is widely accepted that Watson excluded consciousness from the scope of psychology. In fact, in Watson (1913a)'s “manifesto,” he stated that consciousness plays no role in behaviorist psychology:

The time seems to have come when psychology must discard all reference to consciousness; when it need no longer delude itself into thinking that it is making mental states the object of observation. We have become so enmeshed in speculative questions concerning the elements of mind, the nature of conscious content (e.g., imageless thought, attitudes, and Bewusseinslage, etc.) that I, as an experimental student, feel that something is wrong with our premises and the types of problems which develop from them.

(p. 163)

However, almost simultaneously, Watson also discussed a behaviorist approach to images and affections (Watson 1913b). Furthermore, in his later work, Watson explored the idea of understanding the thinking process as subvocal speech (Watson 1924). While these considerations were scientifically unsound, it was an attempt to transcend simply treating behavior as the scientific object of study, moving toward an ontological argument where mental concepts are not hidden processes inaccessible to third‐party observation, but rather behavioral phenomena. This stance is referred to as *metaphysical behaviorism*, and it has been pointed out that Watson was not fully aware of the distinction, as both elements are intertwined in Watsonian behaviorism (Morris and Todd 1999).

The above provides a general characterization of Watsonian Behaviorism. Let us now address Tolman's stance on these features. Tolman agreed with the first claim. For example, Tolman (1926) discusses how behaviorists treat the concept of “idea,” asserting that “We behaviorists, whatever else our divergences, are agreed in viewing overt behavior as the primary datum for psychology” (p. 353). This statement underscores his adherence to the fundamental position of behaviorism. However, Tolman explicitly opposed the second feature of Watsonian behaviorism. He referred to behavior in Watsonian behaviorism as “molecular behavior” and contrasted it with his own research program, which dealt with “molar behavior.” Tolman (1932) defined it as follows.

…behavior, as such, is more than and different from the sum of its physiological parts. Behavior, as such, is an ‘emergent’ phenomenon that has descriptive and defining properties of its own. And we shall define this latter as the molar definition of behavior.

(p.7)

The molar approach to behavior was strongly influenced by Gestalt psychology. The famous slogan “the whole is greater than the sum of its parts” originated from Gestalt psychology. While Gestalt psychology is renowned for applying this concept to perception, it is, in fact, a theoretical framework that spans a wide range of phenomena, including behavior and learning (Koffka 1935/2013).

In response to the third characteristic of Watsonian behaviorism—namely, its exclusion of consciousness and mental concepts—Tolman offered an alternative grounded in his theory of sign‐Gestalt. To clarify this theory, it is helpful to first contrast it with the notion of Gestalt in Gestalt psychology. In that framework, the basic unit of perception is not an aggregate of discrete sensations but a structured whole. When organisms perceive an object, they do not assemble it from isolated parts; rather, they perceive it as a unified figure against a background—a phenomenon known as figure‐ground organization. This irreducible wholeness is what Gestalt theory calls a “Gestalt,” and it is considered a prerequisite for meaningful perception. Building on this foundation, Tolman extended the Gestalt perspective beyond perception to the level of behavior. His sign‐Gestalt incorporates not only perceptual elements but also goal‐directed relations, such as the connection between a stimulus, an anticipated outcome, and behavior as a means to achieve it.

Sign‐Gestalt psychology took an additional step by asserting that organisms do not merely perceive; they also “propositionalize” the object (Tolman 1933a). In this framework, the figure and ground concept from Gestalt psychology is perceived within a larger whole. This whole is the sign‐Gestalt, which encompasses signs, significates, and means‐end relations. Thus, in the sign‐Gestalt perspective, the process begins with the formation of a perceptual unit, the sign‐object, derived from sensory input, much like in Gestalt psychology. However, beyond this, the target object of the action, referred to as the signified object, and the means‐end relationship between them are simultaneously perceived. In other words, the “whole” in sign‐Gestalt includes not only the object but also the potential actions associated with it. Tolman (1933a) refers to this position as “propositionalism.”

…visual stimuli will be thought to evoke not just this configuration as a mere ‘perceived’ given, but rather some specific larger whole in which this merely pictured (i.e., perceived) configuration will itself be embedded as one term in a larger means‐end ‘proposition’ such, as that: “this chair, if sat on, will lead to rest (or its reverse).

(pp. 394–395)

The formation of a sign‐Gestalt refers to the idea that what is perceived is a whole that can be summarized as a proposition, rather than the external world itself being directly represented as such.

In perceiving the means‐end relation of objects related to action, Tolman distinguished between the sensory properties of an object, which he termed discriminanda, and the properties that enable motor manipulation, which he termed manipulanda. Discriminanda refers to “actual discrimination‐possibilities of the given environment which result from the nature of the environmental object and from the sense‐organ make‐up of the species of individual animal in question” (Tolman 1933a, p. 395‐396). Furthermore, Tolman argued that when animals encounter their actual environment, they do so with a certain “cognitive set,” which he referred to as discriminanda‐expectation. Similarly, while manipulanda are objects in the environment that allow for manipulation, the organism's preparedness for action is referred to as manipulanda‐expectation (Tolman 1932, 1933a).

The emergence of manipulanda‐expectation in the organism is closely related to the separation of the figure from the ground and the establishment of Gestalt. For instance, an object's independence from the background as a distinct entity arises when properties such as the potential to grasp are attributed to the organism. In this sense, manipulanda are “configurating properties” (Tolman 1933a, 400). A sign‐Gestalt is formed through the perception of a whole that includes not only discriminanda and manipulanda but also the object's availability for use (i.e., the means‐end relation). Tolman, based on his research in maze learning, argued that animal learning occurs not only in the trial‐and‐error theory, which was a dominant explanation at the time, but also in the sign‐Gestalt‐type learning process (Tolman 1933b).

It seems worthwhile, at this point, to note explicitly whether Tolman was in fact concerned with perception. In the latter half of this article we examine Tolman's influence on Merleau‐Ponty. Yet in the conventional reception, Merleau‐Ponty is typically located within a tradition of perception, whereas Tolman is placed within a tradition of learning. Because contemporary psychology treats perception and learning as distinct domains, some readers may suspect that this juxtaposition undermines the aims of the present paper. In fact, however, it is already evident from the foregoing discussion that Tolman did attend to perception, insofar as he adopted the sign‐Gestalt as a principle of perceptual organization that guides behavior. Historically speaking, Tolman's sign‐Gestalt theory incorporated an account of organismic perception into a theory of action to a greater extent than did many of his contemporaries. Discrimination, to be sure, had attracted the interest of animal psychologists well before Tolman (e.g., Watson 1909; Yerkes 1912), but it had not yet been developed into a detailed theory of perceptual organization. Tolman's focus on this issue reflects his sense that, if one is to address an organism's purposiveness while maintaining a non‐mentalistic stance, perception must be formulated as an intentionality toward objective features of the environment (Tolman 1934). This perspective drew on Egon Brunswik. Together, Tolman and Brunswik aimed at “combining perception and means‐end action into one picture” (Tolman and Brunswik 1935, 47). On their view, organisms confront a common problem in both perception and goal‐directed action: the process by which the organism becomes coupled to the probabilistic and causal structure of its environment. Taken together, these considerations make it clear that the contemporary division of labor between learning and perception does not provide a strict partition of Tolman's concerns.

## 3 Is Sign‐Gestalt Representational?

Tolman's theory of sign‐Gestalt occupies an ambiguous position regarding the question of mental representation. While it was intended to describe behavior and perception at a level broader than the traditional Gestalt, it remains unclear whether sign‐Gestalt refers to an internal cognitive representation or to an organism's embodied interaction with its environment. This ontological ambiguity was never fully resolved by Tolman himself, and has been a point of critique by contemporaries such as Koffka (1933), who questioned whether Tolman's behaviorist commitments prevented him from properly addressing the internal reality of mental constructs.

Koffka noted Tolman's indecision in definitively labeling the concept as an internal construct, despite his intention to maintain a behaviorist stance. Koffka remarked, “Tolman, I am certain, wants to establish the reality of these molar characteristics. But being a pragmatic behaviorist, he substitutes for these realities mere fictitious constituents of a logical system” (444). Koffka further emphasized that research on internal processes in animals should explicitly address concepts such as purpose, cognition, and expectation. This ambiguity arose because Tolman emphasized the possibility of public observation within behaviorism. For example, Tolman noted that discriminanda‐expectation corresponds to the sensation of introspective psychology at the time, but he preferred terms that could be clearly defined even in animals, insisting on maintaining his position within a behaviorist framework. As a result, it may have become difficult for Tolman to fully engage in a discussion of the ontological status of his concept. Instead, as Delmore (2024) argued, Tolman's concepts function as instrumentalist metaphors, which accounts for their intrinsic ambiguity and the tendency to be interpreted as precursors to cognitivism. For instance, Tolman (1926) stated the following:

We have, then, these two conclusions: (i) Behavior expresses immanent purposes,—purposes which exhibit themselves as persistences through trial and error to get to or from. And (2) it expresses, as is indicated by the facts of learning, immanent cognitions,—cognitions as to the nature of the environment for mediating such gettings to or from. It is these cognitions which are of chief interest to us tonight. They are ideas, at least in some sense of that word.

(p. 356)

In this passage, “purpose” can be interpreted as a construct that can be measured through behavior as a signature, reminding operationism to define mental concepts by observable behavior and its measurement. Likewise, Tolman already engaged in a thinking akin to later operationism when defining emotion behavioristically (Tolman 1923). This argument also involves measuring psychological phenomena that are not publicly observable through alternative behaviors. Since operationalism and the operational definition of related concepts explicitly permeated psychology in the 1930s, Tolman's ideas can be seen as anticipatory of this approach (Hardcastle 1995; Stevens 1935).

However, in his later years, Tolman argued that psychological theory could not be adequately formulated using only intervening variables, and that theorizing should instead incorporate hypothetical constructs (Tolman 1949). The distinction between intervening variables and hypothetical constructs was introduced by MacCorquodale and Meehl (1948). While intervening variables are merely functional expressions defined by the relations between events (e.g., stimulus and response, or testing methods), hypothetical constructs carry a surplus meaning that cannot be fully reduced to such functions. The deliberate use of mental concepts as hypothetical constructs, rather than merely as functional placeholders, was in fact a key aspect of the so‐called “cognitive revolution” (Greenwood 1999). In this sense, later Tolman has been widely received as anticipating a cognitivist perspective. That said, in the earlier stages of his career, Tolman expressed concern that his arguments might be interpreted as overly mentalistic (Tolman 1932, xvii–xviii). This suggests that early on, he did not consider his cognitive concepts to be synonymous with internal mental processes.

In line with our interpretation, Delmore (2024) offers a strong critique of the conventional interpretation that casts Tolman as a precursor to cognitivism. Instead, he situated Tolman within a lineage of psychology irreducible to physiology, tracing back to William James. Tolman's direct mentor, Edwin B. Holt, likewise sought to reconstruct a theory of behavior that took seriously the relational nature between organism and environment (e.g., Holt 1915). Against this intellectual backdrop, Tolman introduced the concept of the cognitive map—not as a hypothesis about internal representations, but as an instrumental model for describing the causal structure of behavior. This model, Delmore argues, should be seen not as an early step toward cognitive science, but rather as part of an internal critique of physiological reductionism within behaviorism itself. It remains within the framework of behaviorism, rather than anticipating its transcendence. We take this to be more than a trivial difference, because it leaves room for interpretation concerning the ontological status of his notion of cognition. As we will see in the following section, it is precisely this ambiguity that Merleau‐Ponty criticized, and reinterpreted Tolman's concepts within his own account.

Although early Tolman consolidated his position through behaviorist lines of thought, later cognitivists came to describe him as their precursor. Was this merely a misreading? Given that Tolman's early project functioned as a remedy for internal problems within the behaviorist framework, interpreting terms such as “purpose,” “expectation,” and “cognition” through the lens of contemporary cognitive psychology is anachronistic. Yet, once two points are kept in view, it becomes clear that Tolman's own ambiguity and intellectual shifts also helped to invite cognitivist readings. First, there is the ambiguity inherent in his prose. Even if Tolman built his position under the guidance of behaviorist motivations and concerns, his papers do contain formulations that later psychologists could plausibly read in cognitive terms. Let us offer a few examples.

(1) the organism may merely ‘feel’ whether these represented results are ones which if actually presented would themselves, given the larger act, lead on to still further behavior. Or (2) if he is of a superior type, he may even represent the results of still further behavior which might lead from these first represented results; and so on through a whole chain of successive representations.

(Tolman 1928, 436)

“To make an adjustment to an act is to achieve a representation (based, of course, upon what has happened upon previous occasions when this act or similar ones have actually been performed) of the probable stimulus‐ results to be expected from the act.”

(Tolman 1927, 436)

It is even more pronounced in Tolman's late‐career writing.

[…] we assert that the central office itself is far more like a map control room than it is like an old‐fashioned telephone exchange. […] the incoming impulses are usually worked over and elaborated in the central control room into a tentative, cognitive‐like map of the environment. And it is this tentative map, indicating routes and paths and environmental relationships, which finally determines what responses, if any, the animal will finally release.

(Tolman 1949, 192)

Psychologists after the rise of cognitive science might find it hard to resist reading cognitivist implications into these passages, retrospectively and with the benefit of hindsight.

Second, there is the historical trajectory of Tolman's own stance. From the mid‐1930s onward, Tolman explicitly adopted operationalism (e.g., Tolman 1937). Operationalism, or the operational definition of concepts, is a procedure for fixing meaning by identifying unobservable mental variables or states with the operations by which they are measured. Despite its well‐known philosophical deficiencies (Leahey 1980; Grace 2001), contemporary psychology has made this move a routine practice in defining psychological variables and cognitive concepts. Tolman's turn to operationalism may therefore have given later champions of cognitive science ample reason to view him as someone who had early recognized the possibility of an objective study of mental concepts. In fact, he shifted his philosophical footing from new realism to operationalism across his early and middle career (Carroll 2017; see also Section 7), but this is a biographical trajectory that most psychologists do not typically have in view. Given these circumstances, a certain degree of ambiguity and confusion in Tolman's claims and their reception might be inevitable.

In summary, Tolman developed his purposive behaviorism as an alternative to Watsonian behaviorism, drawing on and reformulating concepts from Gestalt psychology in the process. Central to this theoretical framework was the idea of sign‐Gestalts, which emphasized that organismic behavior does not arise in a one‐to‐one correspondence with discrete stimuli, but rather emerges from a process of structuring the environment as a whole. Tolman's early writings remained ambiguous about whether the processes he described involved the manipulation of internal representations. Nevertheless, his eventual adoption of hypothetical constructs rendered his framework more compatible with cognitive science. Consequently, his key concepts have often been retrospectively interpreted in representational terms. Notably, however, Merleau‐Ponty did not inherit Tolman's concepts in a representationalist manner.

## 4 Tolman's Influence on Merleau‐Ponty

Merleau‐Ponty engages with Tolman's notion's in his early work, *The Structure of Behavior* (Merleau‐Ponty 1963; French original 1942). Rather than simply rejecting or adopting Tolman's ideas, he developed his philosophy based on them. That is, Tolman's sign‐Gestalt theory worked as a catalyst for clarifying and refining Merleau‐Ponty's own philosophical perspective on behavior by discerning ambiguity of Tolman. Merleau‐Ponty's aim in *The Structure of Behavior* was, as he puts it, “to understand the relations of consciousness and nature: organic, psychological or even social” (Merleau‐Ponty 1963, 3). This section examines how Merleau‐Ponty drew on Tolman's thought to articulate a view of behavior as structurally meaningful, yet non‐representational, and how this reinterpretation shaped his broader philosophical project.

Before turning to Tolman's specific influence, it is worth situating *The Structure of Behavior* within his oeuvre. This major early works was submitted as the complementary thesis for his doctorate and published in 1942, alongside what would later appear as *Phenomenology of Perception* (Merleau‐Ponty 1996 French original 1945). Although Merleau‐Ponty is generally regarded as a phenomenologist, *The Structure of Behavior* makes sparing use of phenomenological concepts and methodologies. Instead, he extensively referenced the contemporary knowledge from psychology and physics, drawing on that literature and, at times, criticizing it in order to develop his own position.

In *The Structure of Behavior*, Merleau‐Ponty reconsidered the relation between consciousness and nature. That is, how the subject of cognition and action, namely mind, relates to its environment. To address this problem, he sought to overcome the traditional opposition between naturalism and intellectualism. He rejected both the reduction of the mind to physical phenomena and, conversely, the reduction of the environment to mental phenomena. Instead, he put forth the idea that the mind and environment are integrated through “behavior.” In this context, behavior was characterized as an intermediary between mind and environment, possessing physical and motoric aspects while essentially having a mental structure (“the structure of behavior”). Merleau‐Ponty invoked to Tolman precisely when articulating this structure, treating him as a precursor for his own account.

It is often said that Merleau‐Ponty criticized behaviorism in *The Structure of Behavior*, and indeed, such criticisms appeared throughout the book. On that basis, it seems unlikely that he could have been influenced by Tolman, who is broadly classified as a behaviorist. A closer reading, however, reveals that Merleau‐Ponty's target was not behaviorism as such, but Watsonian behaviorism in particular (e.g., Merleau‐Ponty 1963, 182). He clearly distinguished Watson from Tolman, and criticized the former's naturalistic orientation, especially, the “conditioned reflex doctrine” that reduces behavior to the mere reception of stimuli and the elicitation of response. As discussed in the previous section, it was precisely this kind of reductionist view that Tolman opposed.

Moreover, Merleau‐Ponty was quite familiar with Tolman's own work and referred to it favorably in his writings. We will discuss the particular features of Tolman's thought that Merleau‐Ponty took up below; for now, it is remarkable that Merleau‐Ponty did not treat Tolman just as a behaviorist. More specifically, he highlighted Tolman's rat experiment as evidence that behavior involves the perception of a meaningful whole where “favorable outcome” and the stimulus constitute a single Gestalt. Merleau‐Ponty remarked favorably on this and, moreover, contrasted Tolman's position with that of the “strict behaviorists.” Merleau‐Ponty's criticism targeted only a narrow strand which leaves open the possibility that he constructively engaged with other, more nuanced behaviorist thinkers such as Tolman. Dismissing Tolman's influence simply because of his association with behaviorism would therefore be misleading.

Although Merleau‐Ponty did not fully endorse Tolman's view, he nonetheless adopted key aspects. What, then, did Merleau‐Ponty inherit from Tolman, and how did Merleau‐Ponty refine his own thought through critique? The following section examines how Tolman's concept shaped core claims of *The Structure of Behavior*. Merleau‐Ponty proposed that behavior is precisely understood within relations of consciousness and nature. He states, for example:

Since all the movements of the organism are always conditioned by external influences, one can, if one wishes, readily treat behavior as an effect of the milieu. But in the same way, since all the stimulations which the organism receives have in turn been possible only by its preceding movements which have culminated in exposing the receptor organ to the external influences, one could also say that the behavior is the first cause of all the stimulations.

(Merleau‐Ponty 1963, 13)

This passage means that behavior is shaped by external stimuli in the environment, while also transforming the environment in which the organism is situated. In this sense, behavior is inseparable from both the subject and the environment; it is not abstract movement that can be isolated from context. Precisely because behavior unifies subject and environment, Merleau‐Ponty characterized behavior as simultaneously “mental” and “physiological,” a single phenomenon grounded in the dynamic interplay between organism and world.

In this respect, behavior is “mental” as well. Merleau‐Ponty therefore treated behavior as a “form,” in opposition to reflex theory and the “strict behaviorism” built upon it (Merleau‐Ponty 1963, 130). The opposed approaches treat behavior as a sequence of independent, separable elements—stimulus and response—whose relations are merely external. In contrast, Merleau‐Ponty emphasized the intrinsic unity of these elements. According to his definition, a Gestalt refers to “total processes whose properties are not the sum of those which the isolated parts would possess”; “total processes which may be indiscernible from each other while their “parts,” compared to each other, differ in absolute size”; and “transposable wholes” (Merleau‐Ponty 1963, 47), in which elements are internally related rather than merely juxtaposed. As long as this internal structure is preserved, the Gestalt remains “transposable”: it can be shifted or recontextualized without losing its identity. However, even a slight disruption in the internal relationships can transform the whole into an entirely different form.

It is important to emphasize that Gestalt does not refer as disordered collection of elements. For Merleau‐Ponty, behavior—the relationship between consciousness and nature—is a form that has structure, just as the title *The Structure of Behavior* suggests:

For instance, the excitations received on the sensory terminations and the movements executed by the effector muscles are integrated into **structures** which play a regulating role in their regard. These **structural processes** account for the laws of learning which we formulated above: since they establish a relation of meaning between situation and response, they explain the fixation of adapted responses and the generality of the acquired aptitude. In the stimulus‐response schema, they bring into play not the material properties of the stimuli, but the formal properties of the situation: the spatial, temporal, numerical and functional relations which are its armature.

(Merleau‐Ponty 1963, 103)

As the term “structure” indicates, the elements within a Gestalt do not exist in isolation; they refer to and influence one another, forming an internally coherent order. The unity of the whole, then, arises not from the elements themselves, but from the dynamic interrelations that constitute them as a system.

Merleau‐Ponty characterized the “structure” of behavior, that is, the relations among its elements, especially between the whole situation and the organism's reaction as “relations of meaning.” These are not chemical or physical relations, but the spatial, temporal, numerical and functional relations established by the subject itself (Merleau‐Ponty 1963, 13). In this sense, they are “mental,” while, for Merleau‐Ponty, the “mental,” never implied isolation from something physical; it instead designated a unity with the environment. Furthermore, he interpreted “meaning” in behavior as a kind of “purposiveness” (Merleau‐Ponty 1963, 49) or, in other words, a relationship of “means to a certain end” (p. 110). Namely, a situation and its constituent elements guide the subject towards specific action. Because of this purposive character, behavior is not only perceived by the conscious subject, but also actively involves the conscious subject. By describing behavior as a form, Merleau‐Ponty aimed to show that once a means‐end relation is perceived in a given situation, the subject is already engaged in that behavioral structure. While we have seen that the structure unifies the elements of action, here we can say more: it also binds the conscious subject and the situation into single and meaningful whole.

In more detail, Merleau‐Ponty distinguished the structure of behavior into three stages: “syncretic forms,” “amovable forms,” and “symbolic forms,” and analyzed each in contemporary psychological findings. In discussing amovable forms, he drew on Tolman's concept of sign‐Gestalt. Here, the agent identifies certain stimuli in the surrounding situation as signals and acts on them accordingly. Unlike syncretic forms, where such responsiveness is governed by instinct, the amovable forms involve signals that are acquired through learning. Merleau‐Ponty suggested that, in its amovable forms, behavior is guided by signals rooted in the “structure of the ensemble,” which is shaped through learning about the environment:

In signal behavior, the “situation” to which the organism adapts is the simple temporal or spatial contiguity of a conditioned and an unconditioned stimulus. But, as we indicated above and must now show, signal learning is not a simple transfer of this *de facto* contiguity into behavior. It must become a contiguity “for the organism.” If spatial contiguity is involved, the unconditioned stimulus is not linked to the conditioned stimulus which is the object of the training, but to a structure of the ensemble of which it is only a moment and from which it derives its meaning […] Thus, the relation of the conditioned stimulus to the conditioned response is a relation between relations. The training does not transpose a *de facto* contiguity into behavior. The signal is a configuration (sign‐Gestalt). (Merleau‐Ponty 1963 106)

In amovable forms, a stimulus (i.e., a conditioned stimulus) is received as a signal for another (unconditioned) stimulus. While these two stimuli are in temporal or spatial contiguity, Merleau‐Ponty emphasized that what matter is not the factual contiguity itself, but the way this contiguity is received as meaningful by the organism. In other words, a stimulus becomes a signal only when it is related to a structure of the ensemble of which it is only a moment and from which it derives its meaning. When Merleau‐Ponty stated that “The signal is a configuration,” he highlighted this structural embeddedness. It is in this context that he introduced Tolman's notion of the sign‐Gestalt in order to articulate this core insight.

To reiterate, Merleau‐Ponty attempted to demonstrate the close connection between consciousness and environment in *The Structure of Behavior*. In developing this argument, he characterized both mental and physical. That is, behavior is not merely physical movement, but is organized by a meaningful mental structure. Merleau‐Ponty interpreted this “meaning” as meaning for a conscious subject acting in a given environment, namely as a means‐end relation.

For Merleau‐Ponty, who sought to locate the mental within behavior and to treat behavior as involving both a conscious subject and the environment as a whole, Tolman provided an attractive point of reference. As noted in the Section 2, while Tolman agreed with Watson that behavior is a proper object of scientific inquiry, he insisted on more molar conception of behavior, and rather than merely reducing behavior physiological chains, developed what he called sign‐Gestalt psychology. Tolman aimed to refine behaviorist psychology by offering a more adequate description of organism's behavior. Merleau‐Ponty, by contrast, intended to show how consciousness and the environment are articulated through behavior. Although their aims did not identical, they nevertheless align closely—at least up to this point.

Merleau‐Ponty instead criticized Tolman's account of the status of the structure of behavior. According to him, Tolman considered only stimulus and response as real, while “determinants” and “functional variables” (Merleau‐Ponty 1963, 182), namely structure of behaviors were not granted such ontological status. On this basis, Merleau‐Ponty commented critically that “‘purposive behaviorism’ remains ‘materialistic’” (Ibid.). Here, “materialistic,” although Merleau‐Ponty did not explicate this, does not mean reduction to the physical in a modern scientific sense, but rather that reality if confined to tangible, isolated events such as stimuli and responses, excluding the structure as a legitimate object of study. Thus, in Merleau‐Ponty's reading, for Tolman, behavioral structure was treated as a theoretical construct rather than something that exists in the world. Merleau‐Ponty, structure as a form mediates between the organism and nature, and is neutral to the classical division between the “mental” and the “physiological.” It is not merely internal or representational. If structure were only “mental,” it could not perform this mediating role. Hence, Merleau‐Ponty rejected Tolman's implicit reduction of behavioral structure to a subjective construct in the end.

Thus, we can observe a fundamental ambivalence in Tolman's thought as interpreted in Merleau‐Ponty. On the one hand, in *The Structure of Behavior*, Merleau‐Ponty favorably referred to Tolman's concept of the sign‐Gestalt to articulate his own account of behavior as a form that mediates between the organism and nature. On the other hand, he criticized Tolman for regarding the structure of behavior as mere mental phenomena, thereby failing to acknowledge its ontological reality. In refining this point, Merleau‐Ponty reasserted the real, mediating function of behavioral structure. In this sense, Tolman served as both a positive and critical source for *The Structure of Behavior*. This sets the stage for the next section, where we trace the indirect conceptual lineage from Tolman, through Merleau‐Ponty, to the foundations of enactivism.

## 5 Tolman's Indirect Contribution to Enactivism via Merleau‐Ponty

As stated in the introduction, enactivism refers to a recent development in the philosophy of cognitive science. It was first proposed by Francisco Varela, Evan Thompson, and Eleanor Rosch in their book *The Embodied Mind* (Varela et al. 1991). Enactivism is defined in part by its rejection of representationalism and computationalism as traditionally understood in cognitive science. For these, it draws intellectual inspiration from Merleau‐Ponty's phenomenology. We find, for instance, the following passage in TEM:

We like to consider our journey in this book as a modern continuation of a program of research founded over a generation ago by the French philosopher, Maurice Merleau‐Ponty. By continuation we do not mean a scholarly consideration of Merleau‐Ponty's thought in the context of contemporary cognitive science. we mean, rather, that Merleau‐Ponty's writings have both inspired and guided our orientation here.

(Varela et al. 1991, lxi)

As such, the authors explicitly characterize themselves as successors to Merleau‐Ponty. Here, we do not assess the accuracy of their interpretation of his thought. Indeed, their work does not aim to provide a scholarly exegesis of Merleau‐Ponty's philosophy. Rather, our interest is with what they draw from Merleau‐Ponty and how they appropriate his ideas in developing enactivism.

At first glance, they appear to have drawn key insights about perception and action from *The Structure of Behavior*. Regarding the enactive approach to perception, they explicitly acknowledge: “This approach to perception was in fact among the central insights of the analysis undertaken by Merleau‐Ponty in his early work” (Varela et al. 1991, 173). Immediately following this remark, they include a relatively lengthy quotation from Merleau‐Ponty. The same passage is echoed in Evan Thompson's later work (*Mind in Life*, 2007).

Even these simple observations indicate that early enactivists saw *The Structure of Behavior* as a key source. What, specifically, did they draw from it, and how did they incorporate it into their own framework? Let us begin by briefly examining the relevant passage from *The Structure of Behavior* cited in *The Embodied Mind*.

Since all the movements of the organism are always conditioned by external influences, one can, if one wishes, readily treat behavior as an effect of the milieu. But in the same way, since all the stimulations which the organism receives have in turn been possible only by its preceding movements which have culminated in exposing the receptor organ to external influences, one could also say that *behavior is the first cause of all the stimulations*./Thus the form of the excitant is created by the organism itself, by its proper manner of offering itself to actions from the outside. Doubtless, in order to be able to subsist, it must encounter a certain number of physical and chemical agents in its surroundings. But it is the organism itself—according to the proper nature of its receptors, the thresholds of its nerve centers and the movements of the organs—*which chooses the stimuli in the physical world to which it will be sensitive*. “The environment (*Umwelt*) emerges from the world through the actualization or the being of the organism—[granted that] an organism can exist only if it succeeds in finding in the world an adequate environment.”

(Merleau‐Ponty 1963, 13, italics added by Varela et al. 1991)

In this quotation, Merleau‐Ponty introduced the concept of the form of the excitant as central to describing the relationship between an organism and its environment. While the organism receives stimulation from the environment and responds through action, the nature of the stimulation itself is determined by the organism's behavior. In this sense, both the stimulus and the environment from which it arises are not only shaping the organism, but are also, as the environment for the organism, actively constituted by the organism itself. Put simply, the organism (or the conscious subject) and its environment exist in a mutually constitutive relationship, with the form of the excitant serving as the key mediating concept. This interpretation aligns with the discussion in the previous chapter. Indeed, this passage succinctly captures Merleau‐Ponty's view in *The Structure of Behavior*—namely, the idea of behavior as a Gestalt, and as a structured and structuring activity.

Additionally, the stimulus referred to in this context is not an objective, external stimulus, but one as it is received by the organism—that is, a perceived stimulus. When we consider that this perceived stimulus is shaped by the organism's behavior, it becomes clear that the form of the excitant simultaneously expresses both perceptual and behavioral dimensions. In both philosophy and cognitive science, it is not uncommon to treat perception and action as distinct or even independent systems. Merleau‐Ponty, by contrast, implicitly argued for their inseparability. Perception, understood as the reception or acquisition of a stimulus, necessarily involves an active engagement with the environment. Conversely, whenever an organism engages in any form of action, it inevitably brings about some environmental change that, in turn, presents the organism with a new stimulus, leading a new perception. In this sense, perception and action are inherently co‐constitutive processes.

This unity of perception and action is likewise grounded in Merleau‐Ponty's conception of behavior as a structured activity. As discussed in the previous section, Merleau‐Ponty's notion of behavioral structure presupposes a framework of meaning established by the conscious subject owing to a signal in the amovable form. In other words, the structure of behavior is not independent of the subject's involvement; it incorporates the subject into the activity itself. Yet this structure is also premised on the subject's perceptual engagement with the situation. Thus, the very idea of a structured behavior already entails the unity of perception and action. From this perspective, it becomes apparent that the Varela et al. (1991) did not merely inherit from Merleau‐Ponty the motif of a unified subject and environment; they also drew from his work the motif of a unified perception and action—both of which are grounded in the Gestalt conception of behavior. This point becomes immediately apparent when we examine their description of the “enactive approach to perception,” which they explicitly associate with the quoted passage. For instance, they wrote:

Thus the overall concern of an enactive approach to perception […] is, rather, to determine the common principles or lawful linkages between sensory and motor systems that explain how action can be perceptually guided in a perceiver‐dependent world.

(Varela et al. 1991, 173)

We can also glean from this passage the early enactivists' broader views on perception, action, and the relationship between the organism and its environment. Specifically, phrases such as “lawful linkages between sensory and motor systems” and “action can be perceptually guided” indicate their insistence on the close interconnection between sensory and motor systems. Moreover, their description of the environment as “perceiver‐dependent” underscores the idea that the structure of the environment is determined by the mode of being of the organism itself.

Taken together, Varela et al. (1991)'s emphasis on the unity of the conscious subject and the environment, as well as the integration of perception and action as a single whole, can be traced back to Merleau‐Ponty's conception of behavior as a Gestalt. For example, in the Chapter 9, they return once again to *The Structure of Behavior*, underscoring its continued relevance to their discussion.

[…] we—that is, cognitive scientists—must determine both how the environment constrains the [cognitive] system and how these constraints themselves are specified by the sensorimotor structure of the [cognitive] system (**recall the quotation from Merleau‐Ponty in the previous chapter**). In so doing, we are able to explain how regularities—sensorimotor and environmental—emerge from structural coupling. The research task in cognitive science is to make transparent the mechanisms by which such coupling actually unfolds and thereby how specific regularities arise.

Varela et al. 1991, 206)

Here, Varela et al. reformulated the task of cognitive science in light of Merleau‐Ponty's thought. They proposed that the central aim is to clarify the mutually constitutive and inseparable relationship between the conscious subject and the environment: how environmental stimuli shape the cognitive system, and how these stimuli are, in turn, shaped by a system that integrates perception and action. In short, cognitive science must explain how cognition emerges from this dynamic, reciprocal relationship. Immediately after this, the authors redefine the enactive approach and offer a revised definition of cognition within the enactivist framework.

It is evident that the task of cognitive science, as framed by Varela et al. (1991), rests on two core ideas: the mutually constitutive unity of the subject and the environment, and the inseparability of perception and action within the subject's cognitive system. As we have seen, both ideas can be traced back to the notion of behavior as a Gestalt. In short, one of the foundational ideas of enactivism can be expressed in terms of behavior as Gestalt. This idea originates in *The Structure of Behavior*, and the founding figures of enactivism are fully aware of this lineage. To reiterate, Merleau‐Ponty gained a significant insight into the concept of Gestalt, which was, at least in part, inherited from Tolman.

Merleau‐Ponty's significance is not limited to *The Embodied Mind*. Evan Thompson, one of the co‐authors, later returned to the same ideas in his book *Mind in Life* (Thompson 2007), where he cited the exact same passage from *The Structure of Behavior* that had appeared in Chapter 8 of *The Embodied Mind*. The following excerpt comes immediately before that quotation.

In all animals, neuronal networks establish and maintain a sensorimotor cycle through which what the animal senses depends directly on how it moves, and how it moves depends directly on what it senses. No animal is a mere passive respondent; every animal meets the environment on its own sensorimotor terms. **Merleau‐Ponty recognized this crucial point in his first work,** ***The Structure of Behavior*** **:**

(Thompson 2007, 47)

Clearly, Thompson also emphasized the inseparable, mutually constitutive unity of the sensory and motor systems—what he refers to as a sensorimotor cycle through which what the animal senses depends directly on how it moves, and how it moves depends directly on what it senses. He discussed the relationship between the animal (i.e., the subject) and its environment through this cycle, and explicitly attributes this idea to Merleau‐Ponty's *The Structure of Behavior*. This line of thought echoes the claims made in *The Embodied Mind*. Thompson extracts an additional concept from Merleau‐Ponty's text. Immediately following the quotation, he wrote:

This passage clearly expresses an autonomy perspective. Organisms display patterns of behavior that require us to see them as autonomous.

(Thompson 2007, 48)

“Autonomy” is a central concept in Thompson's formulation of autopoietic enactivism (Ward et al. 2017). It expresses the idea that life emerges through the organism's interactions with its environment, which unfold through perception and action. In *Mind in Life*, then, the notion of behavior as a Gestalt plays a role equal to, if not greater than, the one it played in *The Embodied Mind*.

Thompson explicitly equated his concept of co‐emergence with Merleau‐Ponty's notion of behavioral structure—that is, behavior as a Gestalt. He defined co‐emergence in abstract and general terms: “An emergent process belongs to an ensemble or network of elements, arises spontaneously or self‐organizes from the locally defined and globally constrained or controlled interactions of those elements, and does not belong to any single element” (Thompson 2007, 61). In other words, co‐emergence refers to phenomena that arise not from the mere sum of individual elements, but from the patterns of interaction and relational dynamics among them. As Thompson himself noted, this is precisely what Merleau‐Ponty referred to as a Gestalt. Following Merleau‐Ponty, Thompson emphasized that behavior must be understood as possessing this Gestalt‐like character.

As we have seen, the founders of enactivism inherited and developed the core idea of behavior as a Gestalt from Merleau‐Ponty's *The Structure of Behavior*. This idea served as a foundation for many of their key theoretical insights. Merleau‐Ponty's own conception of behavior as a Gestalt was shaped—both positively and negatively—by Tolman's notion of the sign‐Gestalt. In sum, these observations reveal that Tolman's sign‐Gestalt concept exerted an indirect influence on enactivism through Merleau‐Ponty. In this light, Tolman appears not only as a forerunner of cognitivism, but also—albeit indirectly—as a hidden source of enactivism. In this sense, Tolman is ambivalent.

## 6 Situating the Present Account Within Tolman's Broader Historical Reception

Finally, we turn to the relation between our view on Tolman and his historical standing in contemporary psychology. Our claim, however, is limited in scope: we have argued that Tolman and Merleau‐Ponty were connected in a way that exerted an indirect influence on enactivism. Accordingly, this section does not aim to undertake a comprehensive reassessment of Tolman's overall place in psychology. Rather, our goal is to situate our diagnosis in relation to the broader range of prevailing assessments of Tolman.

In textbook characterizations, Tolman is often presented as a forerunner of cognitivism. For example, Benjamin (2023) labeled Tolman's position “cognitive behaviorism.” In this portrayal, Tolman's use of mental concepts was difficult for behaviorists of his generation to accept, and he was treated as a figure who lost out in his own era and consequently remained neglected until the rise of cognitive science^1^. George Mandler, one of the leading figures of the “cognitive revolution,” likewise cited Tolman as a cognitivist researcher (Mandler 2011). In Howard Gardner's well‐known *The Mind's New Science*, Tolman was depicted as a contradictory figure who, despite betraying the doctrines of behaviorism, nonetheless continued to adhere to them (Gardner 1987).

On our diagnosis, the foregoing view is one‐sided, insofar as it fails to register Tolman's sustained orientation toward a non‐mentalistic explanatory framework. This tendency is especially evident in his early career, during the 1920s and 1930s (Carroll 2017; Delmore 2024). In these periods, Tolman introduced what would later be read as “cognitive” notions not as cognitive processes in the contemporary sense, but primarily as instrumental models. His main target was Watson's molecular, analytic perspective on behavior. As noted in Section 3, the distinctly cognitivist cast of Tolman's concepts emerges with his attitude of treating mental concepts as hypothetical constructs, an assessment he articulated retrospectively when he looked back on his early work late in his career (Tolman 1948). At the same time, even in his early writings one can find scattered formulations that evoke operationalism, as well as passages that can be read as construing cognition as an internal process. For this reason, without a solid grasp of Tolman's own stance, his prose can appear ambiguous, and a cognitivist misreading is, in a sense, understandable^2^.

Historians of psychology have tried to explain why Tolman adopted such an equivocal position. Smith (1986) located Tolman's early career within the lineage of the new realism associated with Edwin Holt and Ralph Perry. What Tolman drew from the new realists was an epistemological stance according to which purposes and expectations are not inner processes of the organism but are instead immanent in behavior. On this view, his position functioned as a two‐front strategy, directed both against the mentalism of William McDougall and against the extreme objectivism of Zing‐Yang Kuo (Carroll 2017, 74–80). In this period, Tolman explicitly rejected the idea that cognitive concepts are “inferred” from behavior (e.g., Tolman 1925, 1928). Even so, there are points at which his prose remains ambiguous, and, as noted above, contemporaries such as Koffka criticized him on precisely these grounds. New realism had already begun to stagnate in the 1920s amid internal tensions and mutual inconsistencies among its proponents (O'Shea 2008). Witnessing this unraveling, Tolman gradually relaxed the claim that purpose and cognition can be read directly off behavior, and eventually revised his view so that the mind came to be treated as something inferred from behavior (Smith 1986). Smith (1986) dates the onset of this transition to 1925 and concludes that Tolman had effectively abandoned new realism by 1935 (p. 89). This decade coincides with the period that we identify as the one in which Tolman's arguments are most readily read as ambiguous and equivocal. In other words, Tolman's apparent ambiguity may reflect a genuine intellectual oscillation between the new realist epistemology that initially oriented his project and his growing mistrust of that framework.

In a sense, Tolman's very ambiguity might leave later researchers a degree of conceptual latitude. As Torracinta (2023) rightly observed, in cognitive science's self‐narratives, the significance of Tolman's work has often been located in its revival of precisely the “mentalism” that Tolman himself rejected, namely, an interest in mentalistic constructs despite his explicit anti‐mentalism. This framing made it possible for early cognitive scientists to cast him as a heroic precursor. Yet, on our diagnosis, the same interpretive openness also enabled a non‐representational appropriation of Tolman's concepts for theorizing perception and action, as in Merleau‐Ponty. If it was this very ambiguity, the ambiguity that permits multiple readings, that licensed the subsequent fertility of Tolman's reception, then clarifying his claims by imposing retrospective hindsight would not yield a more accurate historical characterization of his place. Put differently, ambiguous claims can matter historically precisely in virtue of their ambiguity.

## 7 Conclusion

This paper aims to reevaluate Tolman's place in the history of psychology by demonstrating that his thought served as a precursor to both cognitivism and enactivism. To this end, we first reviewed how his concept of the “sign‐Gestalt” was received as being aligned with core tenets of cognitivism. However, in contrast to this commonly accepted view, the early Merleau‐Ponty developed his own ideas through a close reading of Gestalt psychology including Tolman's work. Merleau‐Ponty's interpretation led to an anti‐representational stance that anticipates key aspects of enactivism.

Accordingly, we demonstrated that one can discern a vague yet proto‐enactive dimension within Tolman's formalization of cognition. Nevertheless, this aspect of Tolman's thought has remained largely implicit to date, arguably because enactivist theorists frequently engage with Merleau‐Ponty while seldom referencing Tolman. When they do, it is often to express categorical rejection against behaviorism. It is important to note, however, that the enactivist critique of behaviorism is primarily directed at Watsonian behaviorism and tends to overlook other figures in the tradition (Yanagawa and Matsui 2025). Tolman's notion of the sign‐Gestalt, in particular, does not warrant such dismissal. Ultimately, acknowledging the ambivalence in Tolman's work opens a space for reinterpreting him as a transitional figure between behaviorism, cognitivism, and enactivism.

## Conflicts of Interest

We have no known conflict of interest to disclose.

## Data Availability

Data sharing not applicable to this article as no datasets were generated or analyzed during the current study.

## Endnotes

1 Even at the level of historical fact, however, this picture is questionable. Tolman's work later gave rise to the Tolman–Hull controversy and, in response, prompted substantial experimental effort (Goodman 2021). This intra‐behaviorist debate also contributed to the discovery of phenomena that remain theoretically important today, such as latent inhibition.

2 As an instance of Tolman's direct influence on early cognitive psychology, Carroll (2017) pointed to George Miller and Jerome Bruner, both of whom were central figures in the emergence of cognitive science. In *Plans and the Structure of Behavior* (Miller et al. 1960), a landmark text of early cognitive psychology, Miller cited Tolman, alongside cybernetics, as a key precursor for modeling how behavior is planned and how it functions as a self‐regulating system. Tolman also maintained personal ties with Bruner and offered advice on *A Study of Thinking* (Bruner et al. 1956). Thus, although Tolman's early work was driven by behaviorist motivations, it is also true that he contributed to the rise of cognitivism.
